# Delirium in German Nursing Homes – a qualitative study of care practice from the perspective of nurses and general practitioners

**DOI:** 10.1186/s12877-026-07592-7

**Published:** 2026-05-05

**Authors:** Maike Kömp, Romy Lauer, Vincent Molitor, Theresa Sophie Busse, Chantal Flemm, Horst Christian Vollmar, Petra Thürmann, Bernhard Holle, Rebecca Palm, Ina Carola Otte

**Affiliations:** 1 Medical Faculty, Department of Health Services Research, Institute for Diversity Medicine, Ruhr University Bochum (RUB), Bochum, Germany; 2 Medical Faculty, Department of Medical Informatics, Biometry and Epidemiology, Ruhr University Bochum (RUB), Bochum, Germany; 3 Medical Faculty, Institute of General Practice and Family Medicine (AM RUB), Ruhr University Bochum (RUB), Bochum, Germany; 4 School VI – School of Medicine and Health Services, Carl von Ossietzky Universität, Oldenburg, Germany; 5 Professorship (Junior) for Digital Health, Department of Human Medicine, Faculty of Health, Witten/Herdecke University, Witten, Germany; 6 Marien Hospital Herne, Department of Geriatric Medicine, Ruhr University Bochum (RUB), Herne, Germany; 7 Faculty of Health, School of Medicine, Chair of Clinical Pharmacology, Witten/Herdecke University, Witten, Germany; 8 Philipp Klee-Institute of Clinical Pharmacology, Helios Hospital Wuppertal, Wuppertal, Germany; 9 German Center of Neurodegenerative Disease – Site Witten, Witten, Germany; 10 Faculty of Health, School of Nursing Science, Witten/Herdecke University, Witten, Germany

**Keywords:** Delirium, Nursing Homes, Health Personnel, Qualitative Research, Long-Term Care, Nurses, General Practitioners

## Abstract

**Background:**

Delirium is a multifactorial and potentially life-threatening syndrome that remains underdiagnosed and undertreated in nursing homes, despite residents’ high vulnerability due to advanced age, multimorbidity and cognitive impairment. To date, little is known how healthcare professionals perceive and manage delirium in these settings, particularly regarding prevention, diagnosis, therapy and interdisciplinary collaboration, as well as differentiation from other neurodegenerative diseases. This study explores the perspectives of nurses and general practitioners (GPs) on the quality of delirium care in German nursing homes in order to identify barriers and opportunities for improvement.

**Methods:**

An exploratory qualitative design was employed. A total of 30 semi-structured interviews were conducted with 15 nurses and 15 GPs in Germany. Participants were recruited using a criterion-based purposive sampling strategy to ensure their direct involvement in nursing home care. Data were collected using collaboratively developed interview guides and analyzed using qualitative content analysis with a deductive-inductive approach.

**Results:**

Both nurses and GPs reported uncertainty and variability in the understanding, recognition and management of delirium in nursing homes. While preventive and other non-pharmacological measures were applied intuitively, they were rarely identified as delirium-specific. Both professions highlighted limited knowledge and training, unclear responsibilities and the absence of standardized tools as major barriers to effective care. Diagnostic practices were largely based on clinical impression rather than structured assessments. Interprofessional and interdisciplinary cooperation was considered essential but was often hindered by organizational factors and individual attitudes. Participants also emphasized the value of involving relatives and other significant others in the care process but noted that this was inconsistent.

**Conclusion:**

Delirium care in German nursing homes is non-standardized and marked by substantial variability in practice and outcomes. Although individual nurses and GPs recognize the challenges and apply some effective routines intuitively, care remains insufficiently systematic and rarely guided by standardized strategies in general. Addressing knowledge gaps, improving interprofessional communication and implementing structured care pathways are crucial steps toward enhancing the prevention, diagnosis and therapy of delirium in these settings.

**Supplementary Information:**

The online version contains supplementary material available at 10.1186/s12877-026-07592-7.

## Background

Delirium is a multifactorial and potentially life-threatening syndrome characterized by acute onset, fluctuating course and impairments in consciousness, attention and cognitive functions. The symptoms cannot be explained by a pre-existing or evolving neurocognitive disorder (e.g. dementia) but are instead attributable to the physiological consequences of a medical illness, substance-related factors, pharmacological effects or multifactorial etiologies [1]. Its clinical presentation is highly variable and may include a broad spectrum of symptoms and underlying causes [2]. This is evident from the fact that delirium can be present in three distinct subtypes: hyperactive, hypoactive and mixed, each with characteristic features that make timely recognition and diagnosis particularly challenging [3]. Hyperactive delirium involves agitation and restlessness; hypoactive delirium shows apathy and lethargy and mixed subtype exhibits fluctuating symptoms of both [1]. This heterogeneity complicates timely recognition and diagnosis [3]. Moreover, delirium often presents with symptoms that overlap with those of neurodegenerative conditions, such as dementia. This makes it particularly challenging to make a differential diagnosis. This symptom overlaps can obscure the acute nature of delirium, leading to its misattribution as pre-existing cognitive impairment [4]. If left untreated, delirium is considered a life-threatening condition. It can lead to hospital admissions or prolonged hospital stays, increased risk of falls, greater need for care, loss of independence, acceleration of pre-existing cognitive decline, and higher mortality rates [5].

Previous research has focused predominantly on delirium care within hospital settings. In contrast, evidence from nursing homes remains limited, available prevalence data on delirium vary widely depending on the setting, with studies in nursing homes from a narrative review reporting rates ranging from 1.4% to 70.3% [6]. These discrepancies may be attributed to differences in study design, diagnostic criteria and characteristics of the study population. Existing research on care practices for delirium in nursing homes is predominantly quantitative, with qualitative insights remaining limited or absent. This study aims to address this gap.

### Delirium in nursing homes: challenges in daily care practice

Residents of nursing homes represent a particularly vulnerable population due to the high prevalence of risk factors such as advanced age, polypharmacy and pre-existing neurodegenerative diseases such as dementia [7]. These factors increase the likelihood of developing delirium, especially in the presence of additional stressors such as infections or medication changes [5].

Despite the serious health risks associated with delirium, it is frequently unrecognized in this setting [8]. As a result, delirium is frequently overlooked in nursing homes because of limited clinical awareness and insufficient diagnostic routines [9]. Increasing evidence suggests that deficits in the recognition, prevention and treatment of delirium in these settings are largely due to knowledge gaps and limited training among general practitioners (GPs) and nursing staff [10, 11]. Moreover, the perspectives of nursing staff and GPs, who play a key role in recognizing and managing delirium, have been scarcely investigated. A deeper understanding of their practical challenges, routines and assessments is essential for developing targeted interventions. Effective delirium care requires early recognition, interprofessional and interdisciplinary cooperation and the implementation of evidence-based management strategies [12].

### DeliA – Delirium in nursing homes

This study is part of the project *DeliA – Delirium in Nursing Homes* (https://delia.info/), funded by the German Innovation Committee of the Federal Joint Committee (G-BA), grant number 01VSF20003. The overarching objective of the project is to determine the prevalence of delirium among residents of nursing homes in Germany, assess the perceptions of GPs and nursing staff regarding the current quality of care, and develop and pilot an interprofessional, interactive e-learning tool for the diagnosis and management of delirium in nursing homes. The study involves experts from multiple disciplines, including nursing, general practice, pharmacology, pharmacy, sociology and health sciences. While participants recruitment and interviews focused on nurses and GPs, the interdisciplinary team informed the study design, the development of the interview guides and vignette and the analytic interpretation of the data.

### Objectives

This study explores how nurses and GPs perceive the current quality of delirium care in nursing homes, with a focus on prevention, diagnosis and therapy. Furthermore, it aims to describe the care situation of individuals with an increased likelihood or active phase of delirium, based on the experiences of nurses and GPs, identifying key barriers to care and highlighting opportunities for improvement from the perspective of healthcare professionals.

Comparable data is not yet available, so the field needs to be analyzed empirically.

## Methods

The results are published in accordance with the standards of the COREQ (Consolidated criteria for reporting qualitative research) checklist [13].

Prior to commencement the positive vote from the ethics committee of Witten/Herdecke University, Germany (application number: 82/2023) was obtained. All participants provided their written informed consent and received compensation for their expenses.

### Design

This study uses an exploratory qualitative research design to examine the quality of current delirium care practice in nursing homes as perceived by nurses and GPs. To ensure the quality and focus of the data, the study design was narrowed to include nursing home settings, two central actor groups (nurses and GPs) and specific aspects of care, such as potential knowledge deficits and communication barriers.

### Data collection

To support an open and participant-centered research process, two semi-structured interview guides (one for nurses and one for GPs) were developed. The development followed an iterative and interdisciplinary process involving all consortium partners and was informed by study objectives, prior project findings and existing literature on delirium care and interprofessional collaboration. The guides were designed to enable participants to share their everyday professional experiences in their own words. The interview guides were adapted for both nurses and GPs to differentiate between the professions (Appendices A and B). While the guides provided a general structure for the interviews, follow-up questions targeted by the interviewers enabled deeper exploration of issues relevant to the research focus. Additionally, field notes were taken during the interviews to capture contextual details and non-verbal cues. They were not pilot-tested and were not provided to participants in advance, in order to encourage spontaneous and experience-based responses.

The content of the interview guides included questions about experiences with the recognition, prevention and treatment of delirium, the use of screening instruments, interprofessional cooperation and the involvement of relatives and other significant others. Each interview began with a short case vignette. The vignette was developed based on findings from a prior realist review within the same research project [14] and was intended to represent a realistic and clinically plausible scenario of delirium in nursing home care. To minimize potential anchoring effects, the vignette was formulated in a neutral manner. Participants were explicitly encouraged to draw on their own professional experiences during the interview and the vignette functioned primarily as an entry point rather than a restrictive frame for the discussion. The use of this vignette was informed by findings from a prior realist review conducted within the same research project [14], which suggests that delirium-related questions may be too abstract or complex without a concrete point of reference. Additionally, two written questionnaires were administered to collect sociodemographic and professional biographical data (Appendices C and D). The questionnaires captured information on personal characteristics, professional role and specialization, length of professional experience and previous experience in the care of residents with delirium.

A female post-doc researcher (RL) employed full-time at Ruhr University Bochum (RUB) (Institute for General and Family Medicine; AM RUB) as a research assistant, who had prior experience in conducting qualitative interviews, conducted the interviews. No prior relationship was established between the interviewer and the participants. Before the interviews took place, the participants were informed about the interviewer’s professional background and institutional affiliation, as well as the aims of the study. Throughout the research process, the interviewer reflected on her disciplinary position and potential preconceptions to minimize bias. The participants were interviewed individually in various settings, such as their homes or workplaces via video conferencing software (Zoom version 6.0) [15] or telephone. For data analysis, all interviews were audio-recorded using a external audio recorder.

### Recruitment

The selection and recruitment of interview participants followed a purposive, criteria-based sampling strategy. Eligible participants were nursing staff and GPs who are involved in the care and support of long-term care residents in German nursing homes. Inclusion criteria were defined as (1) belonging to the professional group of nursing staff or GPs and (2) current involvement in the long-term care of nursing home residents. Nursing homes and GPs were contacted to recruit potential participants. Contacts were made by email, telephone, newsletters, posters and other public information sources, as well as through leaflets and flyers. In addition, existing networks of the Institute of General Practice and Family Medicine (AM RUB) and the North Rhine-Westphalian General Practice Research Network (HAFO.NRW) were used. Publicly available contact details (e.g., from the Association of Statutory Health Insurance Physicians) were also used to contact GPs. Information letters and consent forms were distributed to interested potential participants.

### Data analysis

All interviews were transcribed verbatim and pseudonymized by a transcription agency prior to analysis. The transcripts were not returned to participants for comment or correction. The data were then analyzed via MAXQDA 24 version 24.9.1 [16], based on the qualitative content analysis [17]. Both deductive and inductive categories were formed to analyze the data material. In the initial step, deductive categories were developed based on the structure and the main topics of the interview guides. These were subsequently expanded by inductive categories derived from the actual interview content (see Table 1). All transcripts were systematically coded in close collaboration between two researchers from the study consortium: a female research associate at Ruhr University Bochum (MK) and a male research associate at Carl Ossietzky University Oldenburg (VM) with different disciplinary backgrounds in health sciences and nursing science. The coding process was conducted in close and continuous exchange, with regular discussions to compare coding decisions and interpretations. This interdisciplinary collaboration enabled the integration of multiple analytic perspectives. Any differences in coding were examined and resolved through consensus, contributing to the iterative refinement of the coding framework. Their collaboration contributed to the validation of the coding framework and enhanced intersubjective reliability. The analytic procedure aligns with the exploratory qualitative research design, while allowing for the abstraction of information from the individual cases and its assignment to generalized categories. This approach allows for the systematic identification of cause, effect relationships and facilitates the theoretical generalization of the findings. Participants were not asked to provide feedback on the findings. Interviews were conducted and transcribed in German. Quotes presented in this manuscript were translated into English by the research team and checked for accuracy by a second researcher.

**Table 1:** Codesystem of the qualitative interviews

Main category (*N*/GP)	Subcategories	Description	Total frequencies
Prevention	Barriers, measures, knowledge	Accounts relating to perceived barriers, existing preventive practices and knowledge or awareness of delirium care	140 codes
Diagnosis	Transfer to GPs, barriers, instruments, causes, time, diagnosis, designation, frequency, relation to dementia, symptoms	Accounts relating to delirium and diagnostic practices, including perceived barriers, use of assessment instruments, symptom interpretation, causal attributions and diagnostic decision-making processes	667 codes
Therapy	Non-pharmacological measures, pharmacological measures	Accounts relating to therapeutic approaches to delirium management, including non-pharmacological and pharmacological strategies, treatment decisions and perceived challenges in care practice	145 codes
Vocational training	Self-assessment of one’s competence, prevention, diagnosis, therapy, needs	Accounts relating to participants’ educational experiences, self-perceived competence and perceived training needs regarding delirium prevention, diagnosis and management	193 codes
Involvement of relatives and other significant others	Opportunities, challenges, solutions strategies	Accounts relating to the roles, experiences and perceived challenges associated with the involvement of relatives and significant others in delirium care	215 codes
Interprofessional and interdisciplinary cooperation	Nurses with nurses, Nurses with GPs, external services, external specialists	Accounts relating to experiences, communication practices and perceived challenges in interprofessional and interdisciplinary collaboration in delirium care	537 codes
General challenges	Personnel situation, facilitators, general wishes	Accounts relating to overarching structural, organizational and practice-related challenges influencing delirium care	289 codes

*GP* General Practitioner, *N* Nurses

## Results

A total of 30 semi-structured interviews were conducted with 15 nurses working in different long-term care facilities and 15 GPs who provide consultative care to the residents in different long-term care facilities. The sample size of 30 participants was defined a priori in accordance with the research proposal. During data collection and concurrent analysis, thematic saturation was monitored. In the later stages of interviewing, no new subcategories or substantively novel aspect relevant to the research question emerged, suggesting that sufficient thematic depth had been reached. The interviews were conducted between July 2023 and November 2024, either by telephone or via video conferencing software (Zoom version 6.0) [15] and ranged from 30 to 69 min in duration (mean: 44.86 min).

From the data collected, several overarching themes emerged that describe the quality of delirium care in nursing homes from the perspective of nurses and GPs (1) Awareness and conceptual understanding of delirium, (2) Prevention, (3) Diagnosis, (4) Therapy, (5) Vocational training, (6) Involvements of relatives and significant others, (7) Interprofessional and interdisciplinary cooperation and (8) General challenges and wishes for the future. The frequency of coding shown in Table 1 can give an initial impression of the extent to which the topics were addressed in the interviews. These points provide an overview of the participants’ statements and offer insight into how they assessed the quality of care for delirium patients.

Quotes are assigned with a numerical reference and a professional group (N=nurse, GP= general practitioners) in the following.

### Sociodemographic and professional biographical data

Sociodemographic characteristics and relevant professional background information were collected using two questionnaires tailored to each professional group. An overview of the participants’ characteristics is presented in Table 2.

**Table 2:** Sociodemographic and professional biographical data

Characteristics of Nurses	Nurses (*n* = 15)	Characteristics of GPs	GPs (*n* = 15)
Gender		Gender	
Female	13	Female	4
Male	2	Male	11
Diverse	0	Diverse	0
Age (range; years)	25–58	Age (range; years)	39–68
Workplace		Workplace	
Nursing Home	14	Solo practice	8
Temporary employment agency	1	Group practice	7
Profession^1^		Specialist medical training^1^	
Nursing service management	4	General practice	8
Residential area management	4	Anesthesiology	1
		Internal medicine	8
Certified nurse	7	Psychiatry	1
		Pulmonology	1
Vocational training / university degree^1^		Years of professional experience (range (mean))	2–42 (22.1)
Nursing training	5		
Geriatric nursing training	10		
Specialized training^1^		Workload percentage (%)	(mean: 98.33)
Wound care specialist (ICW-certified)	2	100	14
		75	1
Pain management nurse	1	Total number of patients per quarter (median (IQR))	mean: 1753.33 1500 (100)
Palliative Care	3	Number of facilities served per quarter (median (IQR))	mean: 4.73 5 (3)
Nurse Unit Manager	2	Number of visits per facility per quarter (median (IQR))	mean: 50.9 9.5 (9)
Nursing Service Management	6	No response	5
Clinical practice instructor	3	Number of patients per facility per quarter (median(IQR))	mean: 40.33 20 (70)
Quality management	2	Number of patients with delirium per quarter (median(IQR))	mean: 13.1 9 (17)
Infection control	1	No response	5
No response	3		
Years of professional experience (median (IQR))	mean: 16.57 18 (11)		
Years of experience in the current position (median (IQR))	mean: 7.5 7 (6)		
Workload percentage (%)	mean: 87.67		
100	9		
<100	6		
Nursing staff size of the entire facility (range (mean))	32-1000 (118.13)		
No response	4		
Nursing staff size in the respective unit/ward (median (IQR))	mean: 19.3 84 (46)		
No response	2		
Specialization of the unit/ward^1^			
Physical illnesses	1		
Mental health care	4		
Dementia	7		
Palliative care	1		
Cognitive impairment care	1		
No response	6		
Number of residents per shift (median (IQR))	mean: 73.6 9 (19)		
No response	2		
Frequency of observed delirium/acute confusion with behavioural changes			
Never	0		
Rarely	8		
Frequently	6		
Always	0		
No response	1		

^1^Multiple responses possible

The questionnaires recorded important professional and organizational characteristics of the participants. For nursing staff, this included professional qualifications, clinical specializations, staffing structures within their facilities and wards, workload indicators such as the number of residents cared for per shift, and the reported frequency of residents suffering from delirium or acute confusion associated with behavioral changes. For general practitioners, information was collected on the type of practice (solo or group practice), medical specializations, the number of nursing home residents and facilities they cared for, and the frequency of visits to nursing homes, with specific reference to in-person visits. In addition, general practitioners reported on their experiences with residents suffering from delirium.

### Awareness and conceptual understanding of delirium

Participants showed considerable variation in their understanding of and approach to the term ‘delirium’ across the interviews. In everyday practice, the term itself was rarely used, instead, nurses and GPs relied on descriptive phrases based on observed symptoms or presumed causes, such as acute confusion, transient confusion, agitation or disorientation, as well as colloquial terms, commonly used in clinical practice to refer to an acute, usually transient confusional state. Of the fifteen nurses interviewed, only one reported regularly using the term delirium, while the others avoided it altogether or only adopted it after a medical diagnosis had been made. Among GPs, use of the term also varied: several GPs reported using the term regularly in routine communication, whereas others stated that they rarely or never did so. This conceptual ambiguity shaped how participants described and assessed acute changes in residents’ behavior, influencing subsequent steps in prevention, recognition and treatment.

*“My colleagues often talk about states of confusion or changes in behavior*,* but they only use the term ‘delirium’ when it’s used by a doctor or hospital. The employees don’t*,* they really write it down or just say altered mental state.” (N12*,* line 252f.)*

### Prevention

The statements in the interviews clearly demonstrated that participants’ knowledge of delirium varied. This is particularly evident in the area of prevention. Among other things, the nurses and GPs who participated listed various preventive measures. Clearly, these measures are not necessarily delirium specific. The main measures include monitoring fluid intake, monitoring medication, avoiding polypharmacy and avoiding changes to the environment, particularly hospitalization.

*“The best prevention is good personal care in the care facility. Is care taken to ensure that residents have enough to drink and eat*,* that their needs are met and that their emotional and physical needs are met?” (GP12*,* line 162f.)*

The participants revealed that preventive actions are often carried out in everyday practice but are not explicitly recognized or labelled for delirium prevention by the participants themselves. They thus described a discrepancy between actual preventive behavior and participants’ self-assessed professional competence in this area. Neither nurses nor GPs saw themselves as explicitly responsible for delirium prevention. Limited time and staff resources, as well as communication deficits, often were reported to lead to delayed or omitted preventive measures.

*“Perhaps I didn’t feel responsible for prevention.” (GP10*,* line 200f.)*

A reported challenge in daily care is that delirium is typically recognized only once specific symptoms are present. Participants described how this distinguished between initiating preventive measures and the recognition of the condition.

The transition between prevention and diagnosis is often perceived as blurred and difficult to delineate by the participants. In addition to individual uncertainties, the interviews highlighted organizational barriers such as unclear responsibilities, limited scope for nurses and delayed initiation of appropriate interventions. Structural factors such as staff shortages, time pressure and high turnover were also described as major obstacles.

### Diagnosis

During the interviews, the participants were asked who was most likely to recognize delirium when caring for residents. Both nurses and GPs stated that nurses were most likely to recognize such a condition. This was explained by the fact that nursing staff (including care assistants and support staff) spend the most time with residents and can therefore be more likely to notice subtle changes in their behavior.

*“In other words*,* it’s the person who knows the resident best in the care setting. Therefore*,* nurses who have worked with a resident for a long period of time are the first to notice changes. It doesn’t matter whether they are a care assistant or a nurse.” (N15*,* line 468f.)*

At the same time, participants noted that hypoactive delirium often remained unrecognized, as it was less visible and frequently attributed to dementia, whereas hyperactive delirium was more easily identified due to its disruptive and conspicuous presentation. Although the diagnosis was typically confirmed by the GP in charge, participants reported that other specialists were occasionally consulted, especially in cases where residents were hospitalized.

To assess existing knowledge of delirium, participants were asked to name possible causes. The nurses identified a total of 30 different potential causes, the most common being dehydration, urinary tract infections, changes in medication, hospitalization and environmental changes. The GPs surveyed listed a total of 34 causes. Frequently cited causes included infections, hospitalization, dehydration (including exsiccosis), changes in medication, environmental changes and hyponatraemia.

The majority of participants reported that a diagnosis of delirium had been made without the use of a standardized assessment tool. Instead, recognition was based on clinical impressions, documentation or interprofessional discussion. Although individual instruments and theoretical models such as the 4 ‘A’s Test’ (4AT) [18], the ‘Need-driven Dementia-compromised Behavior Model’ [19], the ‘Mini-Mental Test’ [20] and the ‘Nursing Delirium Scale’ (Nu-DESC) [21] were mentioned, they are not regularly used in practice. There was a general consensus among participants that the implementation of such tools would be beneficial.

*“No*,* I haven’t implemented that yet.” (GP12*,* line 81)*.

*“Now that I think about it*,* I don’t actually have any questionnaires. I’ve never seen a questionnaire before*,* if I’m not mistaken.” (N02*,* line 163f.)*

**Fig. 1:**
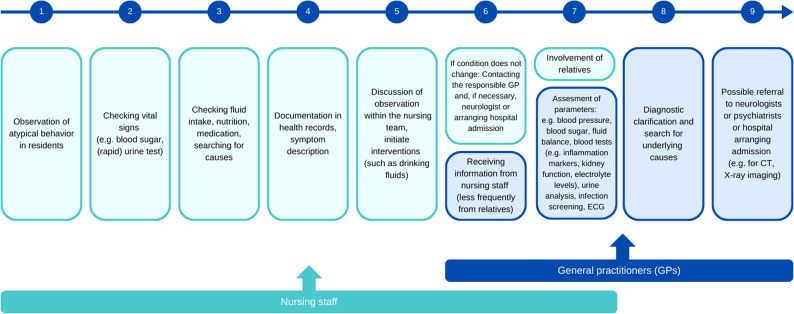
Diagnostic steps (author’s own illustration)

As part of the survey, nurses and GPs provided detailed descriptions of how behavioral changes in nursing home residents are assessed and diagnosed. These statements offer valuable insight into existing care processes and demonstrate how interprofessional collaboration between nurses and GPs is structured in practice. Figure 1 visualizes the diagnostic steps as described by the participants. It summarizes the multi-stage process they reported, combining nursing observations with subsequent medical assessments.

The participants also voiced criticism of the diagnostic process. Primary concerns included the limited authority of nursing staff and structural constraints that hinder timely action.

*“The problem is that you first need to undergo the diagnostic process to receive a delirium diagnosis*,* after which you can act accordingly.” (N03*,* line 410f.)*

Both nurses and GPs described differentiating between delirium and dementia as one of the most challenging aspects of diagnostic practice in nursing homes. Participants reported considerable uncertainty in recognizing acute cognitive changes, particularly in residents with pre-existing dementia. Nurses often said that behavioral changes were initially put down to dementia rather than being seen as possible signs of delirium, especially the hypoactive delirium subtype. GPs emphasized the importance of assessing the time when symptoms first appeared and how they differed from the persons’ usual state, as delirium is characterized by an acute change of state in contrary to dementia. Both groups agreed that dementia substantially increases the risk of delirium while also making it more difficult to recognize.

*“Unfortunately*,* such changes are often downplayed and attributed to dementia-related changes.” (N11*,* line 79f.)*

### Therapy

Both groups of participants perceived the use of non-pharmacological treatments of delirium as time intensive and therefore applied them only to a limited extent.

*“Ultimately*,* we don’t have the time.” (GP09*,* line 344)*.

Participants generally agreed on the importance of such interventions. Nurses emphasized ensuring sufficient hydration, providing environmental orientation, and offering gentle stimulation as core aspects. They also prioritize mobilization through basal stimulation, a basic sensory-oriented nursing intervention, and individualized care routines [22]. GPs also regard basal stimulation, close supervision and careful observation of residents as essential. Participants frequently referred to basal stimulation, despite its absence from current delirium guidelines as a standard intervention. They also emphasized needs-based environmental modifications and social integration as key elements of their delirium care practices.

The interviews described the main criteria for good medication care for people with delirium, such as the regular review of medication plans, the avoidance of drug interactions and polypharmacy and the appropriate management of symptoms. GPs described various drug treatments they use to address the symptoms of delirium. Frequently used medications include melperone, risperidone and promethazine. According to participants, these agents were typically administered in situations of severe agitation, acute distress or when residents posed a risk to themselves or others and were generally considered only after non-pharmacological strategies had been insufficient.

*“Usually for symptomatic treatment*,* depending on whether the individuals are aggressive*,* pose a danger to themselves or others.” (GP11*,* line 153f.)*

Participants’ accounts also indicate that the choice of medication was not always aligned with current delirium guidelines. While melperone is authorized for delirium treatment and risperidone is recommended in some guidelines, promethazine has anticholinergic and antihistaminergic properties that may potentially worsen delirium. However, none of the participants explicitly referred to existing delirium guidelines or mentioned using them to guide pharmacological decisions. For medication reviews, the use of lists containing potentially inappropriate and delirogenic medications for older adults was recommended, such as the PRISCUS list [23]. It has been reported that pharmacists involved in the care of residents are sometimes included in medication reviews. It was emphasized here that it is particularly important to check medication after residents have been admitted to nursing homes or hospitalized. However, according to the professionals interviewed, the residents’ medication plans are often not checked enough.

*“Ultimately*,* the medication plan will only change if the general condition is significantly reduced. You should only consider what is absolutely essential.” (N05*,* line 299f.)*

### Vocational training

Participants described clear deficits in both professional training and perceived competence among nurses and GPs regarding the care of delirium. Both groups consistently reported that delirium received little to no attention during their initial education or training.

Nurses predominantly stated that the topic of delirium was barely addressed during their vocational training, with only a few having encountered more in-depth content through specialized seminars (e.g. advanced training programs for nurses specializing in gerontopsychiatric care). Self-assessed competence varied: In the area of prevention, many expressed uncertainties, although specific individual measures (e.g., monitoring fluid intake, close observation) were cited as strengths. Diagnostic and therapeutic competence were described as limited, with structural constraints and knowledge gaps frequently mentioned.

*“I hardly had to deal with it during my training. Delirium was only briefly mentioned in my further training.” (N11*,* line 316f.)*

Similarly, GPs reported that delirium was inadequately covered during medical school and GP residency, noting that they had encountered the term primarily during hospital rotations. Relevant content was more commonly encountered in geriatric or psychiatric specializations. Their self-assessed competence in prevention of delirium is generally low, although collaboration with nursing staff is considered valuable support. Diagnostic and therapeutic confidence varies greatly and, according to the participants, seems to be highly dependent on individual experience. In addition, it was reported that diagnostic uncertainty is often linked to a lack of clear criteria. In treatment, GPs tend to focus on symptom management or refer residents to specialists.

*“We didn’t learn much about this in our studies. During my specialist training*,* however*,* I worked in a geriatric department*,* where I gained much insight.” (GP13*,* line 138f.)*

Across both professions, a strong need for targeted continuing education and training was expressed. According to the participants, the most important priorities include the establishment of clear diagnostic criteria, structured guidelines (e.g. checklists, assessment tools) and intensified interprofessional collaboration. Integrating delirium more firmly into educational curricula and promoting regular communication between nursing staff and physicians were seen as essential steps toward improving the quality of care.

### Involvement of relatives and significant others

Both nurses and GPs considered the involvement of relatives and significant others as a valuable resource in managing delirium, particularly for prevention, early detection and providing a biographical context. At the same time, they reported challenges such as emotional distress, unrealistic expectations, irregular presence and increased communication demands among relatives and significant others.

*“Many people are also involved and help their relatives. Usually*,* it’s the children or sometimes the grandchildren. They can often provide very good information.“ (GP13*,* line 198f.)*

Structured and regular communication was emphasized as key to avoiding misunderstandings and ensuring constructive and effective involvement. The participants highlighted that relatives or significant others can often identify subtle changes in a resident’s behavior, especially when staff is unfamiliar with the individual or under time pressure. This makes crucial points of reference to them, particularly in facilities where staff turnover is high or documentation is inconsistent. However, several participants also noted that relatives and significant others are not always readily available or present and that expectations about care, especially with respect to pharmacological treatment or hospital transfers, can sometimes conflict with clinical recommendations. Nurses, in particular, stressed the emotional pressure they face when trying to mediate between medical guidelines and family concerns. Although it was reported that relatives or significant others are rarely systematically integrated into care structures, participants suggested that this could be improved through standard communication routines and better education on delirium-related symptoms and risks.

### Interprofessional and interdisciplinary cooperation

The interviews emphasize that effective delirium care depends heavily on well-functioning interprofessional and interdisciplinary collaboration. Nursing staff emphasized the importance of structured communication within the nursing team, particularly through shift handovers and case discussions, to detect behavioral changes at an early stage. Collaboration with non-nursing personnel, such as care assistants or support staff, was seen as potentially valuable, as these groups often notice subtle changes in residents. However, they are rarely included in formal communication structures, which hampers the consolidation of relevant observations.

*“Yes*,* there is cooperation from super good to super bad.” (N07*,* line 288f.)*

The participants made it clear that collaboration between nurses and GPs is shaped by organizational and structural challenges. Communication typically takes place via telephone, email or fax and often depends heavily on the physicians’ availability. According to the nurses interviewed, personal consultations are becoming increasingly rare, with medical assessments frequently taking place remotely or through written recommendations. The organizational model of GP involvement also influenced communication practices. Some nursing homes relied on one dedicated GP or a small group of locally based GPs who were responsible for all residents, which supported continuity and more predictable collaboration. In other facilities, however, residents continued to be treated by their previous GPs after admission, resulting in a heterogeneous group of external physicians with varying levels of availability, familiarity with the nursing home context and responsiveness. Several nurses also emphasized that the quality of collaboration with GPs strongly depended on the physicians’ individual attitudes and willingness to engage with nursing expertise. Participants described communication as noticeably more effective when GPs were open to listening to nurses’ assessments and trusted their familiarity with residents. In contrast, some nurses reported that certain physicians rarely visited the facility, questioned whether they had been contacted appropriately, or dismissed nursing concerns, which further hindered effective cooperation. From the GPs’ perspective, the nurses’ familiarity with residents is considered a key asset in detecting delirium. However, the quality of nursing documentation and verbal handovers is perceived as highly heterogeneous. Experienced and long-standing nurses are especially valued as reliable partners in the recognition and management of acute changes in residents’ conditions.

*“Depending on who’s on shift*,* some of the nurses are highly experienced and have a good sense of what’s going on. You can rely on them to say*,* ‘Something’s not right’.” (GP05*,* line 316f.)*

Both professional groups identified several barriers to effective collaboration, including high workloads, unclear responsibilities, limited interprofessional understanding and staff turnover. These factors hinder both the observation and documentation of resident behavior and the timely initiation of appropriate interventions.

Furthermore, a lack of standardized communication tools (e.g., checklists, structured handover formats) and varying levels of geriatric expertise across team members were described as central challenges. GPs have emphasized the importance of joint structures such as interprofessional case discussions or regular team meetings, which are currently only sporadically implemented.

*“I think it would be interesting to establish structures that would be useful for preventing and detecting delirium early on*,* as well as for standardizing treatment. I believe that these structures can be established only by bringing people together. This brings us back to the importance of cooperation.” (GP10*,* line 349f.)*

In addition to collaboration within nursing homes, participants described considerable challenges in communication with hospitals. Residents were frequently transferred to hospital due to acute confusion or returned with persistent or newly developed delirium, yet information on diagnostic findings, treatment decisions or medication changes was often incomplete or missing. Discharge letters are usually sent to the GP, however the copy for patients could be handed over to the nurses, what does not happen in most cases. This lack of direct communication was perceived as a major barrier to continuity of care, particularly when residents returned with unresolved or unclear symptoms. Nurses also described practical difficulties in reaching hospital staff or obtaining timely updates during a resident’s stay. While some GPs considered communication with hospitals to be relatively straightforward, nursing staff emphasized that missing or delayed information frequently hindered the assessment of residents’ post-hospital condition and the timely recognition of delirium. Overall, participants viewed hospital transitions as a vulnerable phase characterized by fragmented communication and a heightened risk of information loss.

### General challenges and wishes for the future

GPs identified structural and organizational challenges affecting delirium care in nursing homes. Chief among these were staffing issues: high turnover, temporary workers and lack of professional continuity were repeatedly named core problems. GPs emphasized the need for familiar, consistent caregivers to detect subtle behavioral changes, especially in residents without involved relatives and other significant others. They highlighted the importance of committed nursing staff, regular GP visits, improved communication and better integration of other professionals, such as therapists. Training in delirium and the use of standardized tools and innovative care models (e.g., video consultations, interdisciplinary quality circles) were seen as keys to improving care. The GPs also advocated expanding nurses’ diagnostic roles via structured checklists and tools and emphasized the need to strengthen education and interprofessional collaboration. In this context, one GP explicitly referred to the diagnostic processes associated with delirium and the perceived division of responsibilities between the various care providers:

*“I wish for a much better shift of acute diagnostics toward outpatient care.” (GP03*,* line 372)*.

Nursing professionals reported extensive challenges related to time constraints, staff shortages and unclear diagnostic boundaries between delirium and dementia. The detection and management of delirium were reported as often hindered by a lack of time, insufficient training and communication with physicians. Staff expressed concern over increasing care demands, late admissions of highly dependent residents and unclear physician availability on weekends. Economic pressures within care facilities and a perceived lack of societal recognition were also described as negative factors. Many cited staffing shortages, high staff fluctuation and reliance on temporary workers as key barriers to recognizing behavioral changes in residents. Additionally, variability in clinical sensitivity and declining expertise due to generalized training models were noted. Nurses called for better structural conditions, more qualified staff, fixed team assignments, standardized tools (e.g. checklists), additional geriatric specialists and mandatory training for all levels of staff. Improved cooperation with GPs and regular interprofessional case discussions were suggested as ways to enhance care quality.

## Discussion

The findings of this qualitative interview study provide essential insights from nurses and GPs into the current state of delirium care in German nursing homes and highlight deficits in the prevention, diagnosis and therapy of delirium among residents of nursing homes. Eight themes were identified from the interviews: (1) Awareness and conceptual understanding of delirium, (2) Prevention, (3) Diagnosis, (4) Therapy, (5) Vocational training, (6) Involvements of relatives and significant others, (7) Interprofessional and interdisciplinary cooperation and (8) General challenges and wishes for the future.

### Awareness and conceptual understanding of delirium

Studies consistently show that delirium is frequently overlooked in nursing home settings, despite the fact that residents are particularly at risk due to conditions such as multimorbidity, polypharmacy and cognitive impairment [9, 10]. This gap is especially critical given the potential reversibility of delirium when it is identified and addressed in a timely manner [24]. Across the literature, a recurring finding is that acute cognitive or behavioral changes are often wrongly attributed to existing dementia rather than being considered potential signs of delirium. This can lead to diagnostic delays and missed treatment opportunities [4]. This diagnostic overshadowing closely mirrors the patterns observed in this study, in which participants frequently relied on descriptive terms such as ‘confusion’ or ‘agitation’ and rarely used the term ‘delirium’ itself.

Limited familiarity with formal terminology and assessment criteria, which was evident from the participants’ statements, further reinforces this tendency. Previous research has identified low delirium awareness, particularly the absence of shared definitions and conceptual clarity, as a central barrier to effective prevention, early detection and timely management [25]. Similar to earlier studies, the findings of this study suggest that nurses and GPs often depend on experiential knowledge rather than structured diagnostic tools, contributing to inconsistent recognition and clinical uncertainty. Notably, delirium was frequently framed as a condition primarily associated with hospital care rather than with nursing homes. At the same time, the variation in how nurses and GPs understood and used the term delirium reflects broader structural challenges within the nursing home sector, including unclear responsibilities, inconsistent communication pathways and insufficient professional training.

### Prevention

The results show that delirium prevention is often carried out unconsciously and not in accordance with standardized guidelines. This highlights the need for standardized care pathways [10]. This observation aligns with the findings of a recent Delphi study, which established a consensus-based curriculum outlining key competencies for delirium prevention, detection and management in nursing homes, emphasizing structured, interprofessional approaches [26]. Notably, the study highlights that responsibility for delirium prevention in nursing homes lies primarily with nursing staff rather than GPs, which is consistent with other studies on delirium care [26, 27]. Similarly, the National Institute for Health and Care Excellence (NICE) guideline underscores the importance of multicomponent, tailored interventions delivered by a trained multidisciplinary team, including structured risk assessments and explicit preventive strategies [28]. The interviews revealed a lack of a clear distinction between prevention and early detection, reflecting and reinforcing the conceptual uncertainty that characterizes current practice. At the same time, interviews revealed a notable gap between participants’ self-assessed professional competence in delirium prevention and the limited extent to which preventive measures were explicitly and systematically implemented in everyday practice.

### Diagnosis

The findings suggest that in the context of delirium diagnosis, the observations of nursing and non-nursing staff are crucial for the timely detection of subtle behavioural changes. Participants frequently described that diagnosis was rarely supported by standardized assessment tools, which was associated in the interviews with experiences of clinical uncertainty, differing interpretations and communication challenges within interdisciplinary teams. The underutilization of tools such as the 4AT [18], the Nu-DESC [21] or the Interprofessional Assessment of Geriatric Delirium (I-AGeD) [29] may reflect a perceived gap between knowledge and implementation. Similar barriers have been reported internationally, such as conceptual ambiguities, gaps in education and low acceptance of delirium screening tools in routine care [30]. From the perspective of participants’ accounts, these findings may highlight the potential value of more systematic integration of screening instruments into (electronic) documentation systems. Such integration could support mor consistent assessment practices, particularly at admission or in response to clinical changes. Participants’ narratives further indicated that delayed recognition of delirium was perceived as a recurrent challenge, potentially contributing to adverse outcomes such as hospitalization, functional decline or even mortality [5]. This pattern of under-recognition and late diagnosis is consistent with findings on cognitive impairment in nursing home residents more broadly, where studies have shown that even severe cognitive deficits often go undetected and remain undocumented [31].

### Therapy

Therapeutically, both nursing staff and GPs emphasize the value of non-pharmacological interventions, such as environmental adjustments, mobilization and close observation. Participants’ accounts further suggested that the primary therapeutic approach in delirium management is the identification and removal or treatment of underlying causes, such as infections, pain, dehydration or medication-related issues. However, interview narratives indicated that these measures are often not implemented in a structured, deliberate way and were sometimes described as being overshadowed by pharmacological strategies. Participants partly attributed this to time constraints, staffing shortages and lack of clear protocols [32]. This has also been highlighted in the NICE guideline, which emphasizes the need for structured, multicomponent non-pharmacological interventions delivered by a trained team [28]. Participants also referred to practice-based approaches such as basal stimulation, commonly used in practice but not yet established as evidence-based for delirium. Furthermore, the NICE guideline clearly states that pharmacological treatment does not constitute the standard of care for delirium management. Medications are intended solely for short-term symptom control in case of severe distress or risk. In contrast, interview data suggest that pharmacological symptom management is frequently equated with delirium therapy as a whole. Despite general acknowledgment of the value of non-pharmacological strategies, a preference for medication use remains apparent in practice. Current clinical guidelines emphasize that pharmacological treatment should not be considered a standard approach to delirium management. Rather, medication is recommended only in exceptional cases, for instance, when severe agitation or distress poses a risk to the individual or others, and non-pharmacological measures have proven insufficient [28]. Furthermore, GPs mentioned several drugs for treatment of delirium which are neither approved for this indication nor recommended by clinical practice guidelines and which might be even worsen this condition. These accounts may reflect variations or uncertainties in prescribing practices. Participants’ narratives also highlighted challenges related to medication review processes, particularly following hospital admissions or major care transitions, which were perceived as potential risk points for resident safety. This may point to variability in care routines, particularly in the coordination and follow-up of medication management during care transitions.

### Vocational training

Vocational training deficits were frequently described by participants as a relevant factor associated with inconsistent delirium management. Participants’ accounts suggested that educational gaps, including limited formal training and the perceived absence of interdisciplinary approaches, may contribute to knowledge-related uncertainties and experiences of clinical insecurity. These observations may highlight the potential value of more structured and interdisciplinary training approaches [14, 33]. Both nurses and GPs reported limited exposure to the topic during their formal education, which was often linked in the interviews to low self-assessed competence, particularly in diagnosis and therapy [34]. Participants’ narratives further indicated that these perceived educational gaps form a key foundation for the observed knowledge deficits, which appear to underline many of the barriers identified in prevention, diagnosis and therapy. While some individual experience or additional training partially compensated for these gaps, a perceived need for more structured continuing education was evident [14]. Although established diagnostic criteria and evidence-based clinical practice guidelines are available, participants describe that such standards are rarely applied consistently in practice [35]. Similarly, screening tools and therapeutic recommendations outlined in current guidelines were often described as unknown or not routinely used, contributing to variability in diagnostic procedures and treatment decisions. It has been reported that, in practice, diagnostic tools and established criteria are rarely applied consistently. Treatment approaches also lack standardization, which reinforces clinical uncertainty and highlights the need for better communication between professionals [14]. Strengthening vocational curricula and providing targeted, interprofessional training opportunities are therefore essential to improve the quality and consistency of delirium care.

### Involvement of relatives and significant others

The involvement of relatives and significant others was frequently described by participants as an important, yet complex, element of delirium care in nursing homes. Although they were considered a valuable source of information for prevention and early detection, as well as for providing biographical details, participants also reported emotional distress, unrealistic expectations and communication-related disputes when roles and responsibilities were unclear.

A closer examination of the results of this study may suggest that this ambivalence is not solely attributable to the involvement of relatives per se, bit may also be associated with the absence of clearly defined roles, boundaries and communication structures. When expectations regarding responsibilities, prognosis or care goals remained implicit or unaligned, family involvement was often described as being experienced as burdensome rather than supportive. Conversely, when relatives were guided, informed and integrated into care processes in a structured manner, their involvements was frequently described as facilitating delirium management.

This interpretation aligns with the findings of Molitor et al., who similarly emphasized that relatives and significant others represent a crucial resource in delirium care but require clear guidance, education and structured communication to ensure their involvement remains supportive rather than challenging [26]. Taking together, these findings may indicate that the quality and framing of involvement of relatives and significant others, rather than its mere presence, may be particularly relevant for delirium care in nursing homes.

### Interprofessional and interdisciplinary cooperation

Interprofessional and interdisciplinary collaboration was frequently described by participants as a key determinant of quality delirium care but was also perceived as being hindered by structural and organizational barriers. The results are consistent with prior assumption that effective delirium management requires tight coordination among multiple professional groups [36]. This corresponds with the notion of delirium as an inherently multidimensional and interprofessional phenomenon, which may benefit from structured collaboration across disciplines to ensure adequate care [37]. In the German nursing home context, GPs are usually not present on site, which participants described as complicating coordination and increases the reliance on nursing staff. Beyond the general absence of GPs on site, the organizational models through which GP care is delivered may further shape collaborative practices. For example, differences in whether nursing homes work with one designated GP or multiple external GPs may influence continuity, accessibility and familiarity with the care environment. Research indicates that such structural heterogeneity, alongside the lack of standardized models of medical involvement, may hinder interprofessional cooperation and contribute to fragmented care processes [38]. Nurses and GPs alike describe unclear responsibilities, discontinuities in care and insufficient feedback loops, which hinder timely intervention. Notably, experienced nurses were consistently described as central figures in bridging gaps between observations and medical decision-making, which may suggest that informal expertise plays an important role in navigating structural constraints.

### General challenges and wishes for the future

Overall, the findings are broadly consistent with central discussions in delirium research, as participants frequently described that delirium care in nursing homes was not perceived as being systematically established nor consistently implemented. Participants’ accounts further indicate discrepancies in knowledge, perceptions and clinical action between and within professional groups. While delirium is receiving increased attention in academic and clinical discourse, there appears to remain a lack of empirical data on the practical implementation of preventive, diagnostic and therapeutic interventions in nursing home contexts. One potential approach to mitigating these role conflicts and gaps in care may involve the integration of advanced nursing roles, such as Advanced Practice Nurses (APNs), who have demonstrated positive effects on delirium management in hospital settings by coordinating preventive strategies, conducting assessments and supporting interprofessional collaboration [39]. However, participants also emphasized that improvements might also be achievable without introducing new professional roles if existing structures were strengthened. This includes established standardized communication procedures between nurses and GPs, such as structured reporting forms, agreed-upon email templates or fixed subject lines, to ensure that clinically relevant information is transmitted efficiently and recognized as urgent when necessary. Developing such low-threshold, context-adapted tools may represent one pragmatic strategy to enhance coordination and reduce delays in recognition and treatment, particularly given the current constraints of staffing, workload and limited on-site medical presence.

Participants’ accounts and prior literature further suggest that digital solutions may offer additional opportunities to support the information exchange. In Germany, for example, electronic patient records allow discharge letters to be transmitted via a digital portal, which may help address challenges related to information transfer. Another option is the use of teleconsultations. A study from Singapore indicate that teleconsultations can be used to discuss behavioral changes with colleagues or medication adjustments [40]. The focus here was primarily on patients with dementia. Another study showed that geriatric, psychiatric, and palliative care consultations conducted via telemedicine resulted in fewer hospital admissions from nursing homes [41]. Technical advances may provide further possibilities to support processes through digitalization in the future.

### Strengths and limitations

This study provides a nuanced, practice-based understanding of delirium care in nursing homes, reflecting the perspectives of two key professional groups: nurses and GPs. The use of qualitative interviews enabled in-depth insights into real-world practices, perceptions and challenges related to delirium prevention, recognition and care. The inclusion of both nursing staff and GPs allowed for a differentiated, interprofessional perspective that captures the complexity of daily care routines. The broad range of professional experience among participants further enriched the data and helped to illustrate different levels of competence and institutional practice.

However, several methodological limitations should be acknowledged. Firstly, the recruitment process revealed that the term ‘delirium’ is not widely recognized in nursing home practice. Several potential participants declined to take part because they felt they did not know enough about delirium. Even among those who ultimately took part, many initially reported limited familiarity with the concept. This reflects an important contextual barrier, but it also implies a potential selection bias. Those who agreed to participate may have been more engaged, motivated or sensitized to the topic than others, which could limit the diversity of perspectives represented. The use of semi-structured interview guides, while promoting flexibility and participant-driven narratives, led some participants to drift away from the core topic of delirium. As a result, the interviewer had to repeatedly redirect the conversation, which may have affected the depth and focus of specific content areas. In addition, the interview guides were not pilot tested prior to data collection. Although the guides were developed collaboratively within the consortium, prior pilot-testing might have helped to further refine question wording, improve thematic focus and reduce the need for redirection during interviews. Furthermore, the use of a case vignette at the beginning of each interview may have unintentionally influenced the responses of the participants. For example, the focus on urinary tract infection and hydration within the vignette may have directed participants’ attention toward these aspects, potentially narrowing or skewing their responses.

Additionally, the recruitment of participants proved particularly challenging, as both professional groups faced significant time constraints, limiting their availability to take part in the study. Especially the GPs were difficult to recruit: several letters, emails and/or phone calls to a great amount of practices were necessary to recruit 15 participants. Although the sample was intentionally limited and non-random, its size and composition were appropriate for the qualitative design, aligning with the predefined sampling strategy. Thematic saturation was reached during the interview process, suggesting that the sample size was sufficient to capture the central patterns relevant to the research question. Moreover, the interviews predominantly involved more experienced nursing professionals, which may further limit the generalizability of the results. Nevertheless, the criteria-based selection of participants enhances the theoretical transferability of the results to structurally similar long-term care settings. Despite these limitations, the study provides a valuable, practice-oriented contribution to an underexplored area of geriatric care and represents one of the first studies in Germany to address this topic.

## Conclusion

This study provides a practice-based assessment of the current quality of delirium care in German nursing homes from the perspective of nurses and GPs. Both professional groups reported that care for delirium is largely inconsistent and underdeveloped, with significant variability in practice and outcomes. Although participants were aware of the challenges and often recognized existing care as inadequate, this awareness rarely translated into systematic or structured approaches to prevention, diagnosis and therapy. In fact, established protocols or standardized procedures for delirium management were solely mentioned, highlighting a considerable gap in the current care provision.

These findings reveal notable knowledge gaps and communication barriers between nurses and GPs, particularly regarding diagnostic criteria, therapeutic strategies and interprofessional responsibilities. Moreover, some useful care routines, such as hydration management, medication review and close observation of residents, are already applied in practice, although often intuitively and without being explicitly. These insights underscore the urgent need to strengthen professional education, foster interprofessional communication and establish standardized care pathways and collaborative routines to improve the management of delirium in nursing homes.

## Supplementary Information

Supplementary Material 1.

Supplementary Material 2.

Supplementary Material 3.

Supplementary Material 4.

## Data Availability

The datasets generated and analyzed during the current study are available from the corresponding author on reasonable request.

## Abbreviations

AM RUB: Institute of General Practice and Family Medicine at Ruhr University Bochum (German: Abteilung für Allgemeinmedizin Ruhr-Universität Bochum)
APN: Advanced Practice Nurse
COREQ: COnsolidated criteria for REporting Qualitative research
CT: Computed Tomography
ECG: Electrocardiogram
G-BA: German Federal Joint Committee (German: Gemeinsamer Bundesausschuss)
GP: General Practitioner
HAFO.NRW: North Rhine-Westphalian General Practice Research Network (German: Hausärztliches Forschungspraxennetz Nordrhein-Westfalen)
I-AGeD: Interprofessional Assessment of Geriatric Delirium
ICW: German wound care certification body (German: Initiative Chronische Wunden e.V.)
IQR: Interquartile Range
N: Nurses
NICE: National Institute for Health and Care Excellence
Nu-DESC: Nursing Delirium Scale
RUB: Ruhr University Bochum
4AT: 4 A’s Test
