# Supplementary material for: Delirium in German Nursing Homes – a qualitative study of care practice from the perspective of nurses and general practitioners
Source: BMC Geriatr. 2026 May 5;26:634. doi: 10.1186/s12877-026-07592-7 (PMC13141596; doi:10.1186/s12877-026-07592-7)
Supplement: Supplementary file 1 — Supplementary Material 1. [file 12877_2026_7592_MOESM1_ESM.docx]

**DeliA: Delirium in Nursing Homes**

Interview Guide for Nurses

**Introduction**

Thank you very much for taking the time to participate in this interview.

I would first like to briefly introduce the topic. The aim of this interview is to explore your experiences with residents in your facility who show acute states of confusion accompanied by temporary changes in behaviour. In addition, we are also conducting interviews with other nursing professionals and with general practitioners in order to capture their perspectives as well.

We are interested in your personal experiences—there are no right or wrong answers. It is especially important to us that you feel comfortable. This means that if any question feels uncomfortable or you do not wish to answer it, you do not have to. Simply let me know, and we will move on to the next question.

As you read in the documents we sent you, which you returned to us, I will record the conversation with an audio device so that I can listen to it again later and analyse it.

Do you have any questions about this or about the study information and the consent form before we begin?

I will now switch on the recorder.

Thank you for agreeing to participate in this interview as part of the project and for consenting to the audio recording. We have already discussed the project, and you are aware of its background and objectives. In this interview, we would like to learn about your experiences with acute states of confusion and temporary behavioural changes among residents of nursing homes. To illustrate this more concretely, I would like to present a case example:

Please imagine Ms Winter, 83 years old and widowed. She is a resident in your nursing home with a wide variety of interests. She has had no prior memory problems. For the last three days, she has had a confirmed urinary tract infection for which she is receiving medication. Since yesterday, Ms Winter has appeared slowed, drowsy and disoriented in time. She confuses nursing staff with her relatives and shows reduced attention during conversations. She also needs extended periods of rest. Due to her disorientation, she experiences a fall and is transferred to hospital for further evaluation. Her confusion worsens during the hospital stay. Upon her return, Ms Winter appears noticeably altered in her behaviour and spends increasing amounts of time in bed.

**Topic I: Introduction to the topic**

- Have you ever observed similar changes in behaviour among residents?
  - Could you describe a specific example from your daily work?
  - How often do situations like the one I described—or the one you described—occur in your daily practice?
- How do you experience such states of confusion or behavioural changes?
  - Which symptoms do you notice? (e.g. hyperactive and hypoactive delirium)
- These kinds of states are classified under the term delirium (according to the American Psychiatric Association). Do you use the term delirium for such conditions?
  - Which terms do you know?
  - Which terms do you primarily use? (e.g. transient confusion, acute confusion)
  - Which terms do your colleagues tend to use?

**Topic II: Experiences with diagnosis, treatment and prevention of delirium**

- What experience do you have with identifying delirium? (If applicable, refer to the case example.)
- When you notice symptoms, what happens next? What are the next steps?
  - Whom do you inform and how?
  - Is there a standard procedure that you follow? If yes: what does it involve?
- Who formally diagnoses delirium?
  - How does the diagnostic process usually proceed?
- What do you pay attention to when identifying delirium?
  - How do you detect signs of delirium?
  - Do you use instruments designed to identify delirium, such as the CAM (Confusion Assessment Method)?
    - If not: do you know reasons why these tools are not used?
  - How and where do you document observed changes that might indicate delirium (free text vs. structured assessment forms)?
- How confident do you feel in identifying delirium?
  - What contributes to this confidence or lack of confidence?
  - If you feel unsure: what would help you feel more confident?
- To what extent is it possible to distinguish delirium from dementia?
  - What challenges arise in this context?
  - Do you observe symptoms such as changes in memory, alertness or attention in residents with dementia?
    - If yes, how do you handle this?
    - If yes, could you give a brief example?
- What approaches do you use to treat delirium? (If applicable, refer to the case example.)
  - Which pharmacological approaches do you use to treat delirium?
  - Which non-pharmacological approaches do you use?
  - How confident do you feel in treating delirium?
    - What contributes to this confidence or lack of confidence?
    - If you feel unsure: what would help you feel more confident?
- What experience do you have with preventing delirium? (If applicable, refer to the case example.)
  - Which measures do you use for delirium prevention?
  - How well are these measures implemented in practice?
  - How confident do you feel in preventing delirium?
    - What contributes to this confidence or uncertainty?
  - How frequently are residents’ medications reviewed? And by whom?

**Topic III: Training**

- What role did the topic of delirium play in your training or education?
- What would you need in order to feel more confident in preventing, recognising and treating delirium? From whom would you wish to receive this support?

**Topic IV: Cooperation with others**

- How is collaboration with your colleagues structured when caring for residents with delirium? (e.g., communication)
  - Collaboration with nurses
  - Collaboration with nursing assistants / trainees / interns
  - Collaboration with other healthcare providers (e.g., psychologists, physiotherapists)
  - Collaboration with other occupational groups (e.g., cleaning staff)
- How are residents’ relatives involved in the detection, treatment and prevention of delirium?
  - What challenges does the involvement of relatives entail?
  - What are the benefits of involving relatives?
  - How does this affect your work?
- How does collaboration with colleagues outside the facility unfold when residents experience delirium?
  - Collaboration with general practitioners
  - Collaboration with specialists (neurology, psychiatry)
  - Collaboration with staff in hospitals or emergency services
- In your opinion, which professional groups are most likely to notice delirium?
- The staff situation is also of interest in this context. How would you describe the staffing situation at your nursing home?
  - How high do you estimate staff turnover to be at the nursing home where you work?
  - How many experienced nursing staff and how many inexperienced nursing staff are there at your nursing home? Do you notice any differences between them?

**Topic V: Review, outlook & wishes**

When you think about everything we have discussed:

- What challenges do you experience overall in the prevention, detection and treatment of delirium?
- What helps you – in addition to what has already been said – in the prevention, detection and treatment of delirium?

We have now discussed the current situation in detail. Now I would also be interested to hear what you would like to see in the future.

- What could be different/better for the residents? What would you like to see? And from whom?
- What could be different/better for you as a nurse and your colleagues? What would you like to see? And from whom?

**Topic VI: Conclusion**

I have now asked all the questions that are important to us. Is there anything else you would like to discuss that we have not covered yet?

Thank you very much for the interview. Switch off the recording device.

- How was it for you?
- Do you have any questions?

noch ansprechen würden?
