# Supplementary material for: Delirium in German Nursing Homes – a qualitative study of care practice from the perspective of nurses and general practitioners
Source: BMC Geriatr. 2026 May 5;26:634. doi: 10.1186/s12877-026-07592-7 (PMC13141596; doi:10.1186/s12877-026-07592-7)
Supplement: Supplementary file 2 — Supplementary Material 2. [file 12877_2026_7592_MOESM2_ESM.docx]

**DeliA: Delirium in Nursing Homes**

Interview Guide for General Practitioners

**Introduction**

Thank you very much for taking the time to participate in this interview.

I would like to briefly introduce the topic. The aim of this study is to explore the experiences of general practitioners regarding short-term episodes of confusion accompanied by temporary changes in behaviour, commonly referred to as delirium, among residents of nursing homes. We are interested in your experiences with prevention, diagnosis and treatment of such episodes. In addition, we are also interviewing nursing staff from the facilities in order to capture their perspectives on the topic.

We are interested in your personal experiences—there are no right or wrong answers. It is especially important to us that you feel comfortable. This means that if any question feels uncomfortable or you do not wish to answer it, you do not have to. Simply let me know, and we will move on to the next question.

Do you have any questions about this or about the study information and the consent form before we begin?

I will now switch on the recorder.

Thank you for agreeing to participate in this interview as part of the project and for consenting to the audio recording. We have already discussed the project, and you are aware of its background and objectives. In this interview, we would like to learn about your experiences with acute states of confusion and temporary behavioural changes among residents of nursing homes. To illustrate this more concretely, I would like to present a case example:

Please imagine Ms Winter, 83 years old and widowed. She is a resident of a nursing home and one of your patients. Until now, she has had no memory problems. Three days ago, she was diagnosed with a urinary tract infection, which is being treated. The nursing staff contacted you because, since yesterday, Ms Winter appears slowed, drowsy and temporally disoriented. She mistakes staff members for relatives and shows reduced activeness. She also requires excessive rest. Due to her disorientation, she falls and is transferred to hospital on your recommendation. Her disorientation, she falls and is transferred to hospital on your recommendation. Her confusion worsen during her hospital stay. Upon returning to the facility, you visit her and find her markedly changes and increasingly bedridden.

**Topic I: Introduction to the topic**

- Have you encountered similar behavioural changes in residents?
  - Could you describe a concrete example from your practice?
  - How frequently do situations like the one described occur in your daily work?
- How do you perceive such confusion or behavioural changes?
  - Which symptoms do you observe? (hyperactive and hypoactive delirium)
- Such states are classified under the term ‘delirium’ according to the American Psychiatric Association. Do you use the term delirium for these states?
  - What other terms do you know?
  - Which terms do you use?

**Topic II: Experience with the diagnosis, treatment and prevention of delirium**

- What experience do you have with the diagnosis of delirium?
  - Have you ever diagnosed delirium? (If applicable, refer to a case study)
  - What do you look for when recognizing delirium?
    - How do you diagnose delirium?
    - Do you use tools as an assessment instrument to make the diagnosis?
  - To what extent do you implement this in your daily work?
  - How confident do you feel about diagnosing delirium?
    - What leads to this confidence/lack of confidence?
- To what extent is it possible to distinguish delirium from dementia?
  - What challenges does this pose?
  - Do you experience such symptoms (change in cognition, alertness, attention) in residents with dementia?
    - If so, please give me a brief example.
- If symptoms are noticed/ a diagnosis has been made, what happens next?
- What experiences do you have with treating delirium? (refer to case study if applicable)
  - Do you use medication to treat delirium?
  - What about non-pharmacological approaches to treating delirium?
  - To what extent do you implement this in your daily work?
  - How confident do you feel about treating delirium?
    - What leads to this confidence/lack of confidence?
- What experiences do you have with the prevention of delirium? (refer to case study if applicable)
  - Are you familiar with measures for preventing delirium?
  - To what extent do you implement this in your daily work?
  - How confident do you feel about preventing delirium?
    - What leads to this confidence/lack of confidence?

**Topic III: Training**

- How important was the the topic of delirium in your studies?
- How important was the topic of delirium in your specialist training?
- What would you need to feel (even) more confident in the prevention, diagnosis and treatment of delirium?

**Topic IV: Collaboration with third parties**

- How do you collaborate with staff at nursing homes when treating patients with delirium? (e.g. communication)
  - Collaboration with nurses
  - Collaboration with nursing assistants / trainees / interns
  - Collaboration with other care providers (psychologists, physical therapists)
  - Cooperation with other professional groups (cleaners, care workers)

- How do you work with colleagues outside the facility when dealing with patients with delirium?
  - Collaboration with medical specialists (neurology, psychiatry)
  - Collaboration with employees in hospitals / emergency services
- Are there differences in cooperation between suspected delirium and confirmed diagnosis?
- In your opinion, which professional groups are more likely to notice delirium?
  - What happens next? What are the next steps?
- The staffing situation is also crucial in this context. How would you describe the staffing situation in the nursing homes you visit?
- How are residents’ relatives involved in the diagnosis, treatment and prevention of delirium?
  - What barriers does the involvement of relatives entail?
  - What are the facilitating factors associated with involving relatives?
  - What impact does this have on your work?

**Topic IV: Review, outlook and wishes**

When you think about everything we have discussed:

- What barriers do you encounter overall in the prevention, diagnosis and treatment of delirium?
- What factors do you experience as promoting the prevention diagnosis and treatment of delirium?

We have now discussed the current situation in detail. Now I would also be interested to hear how you would like things to be in the future.

- What could be different/better for patients? What would you like to see? And from whom?
- What could be different/better for you as a general practitioner? What would you like to see? And from whom?

**Topic V: Conclusion**

I have now asked all the questions that are important to us. Is there anything else you would like to discuss that we have not covered yet?

Thank you very much for the interview. Turn off the recording device.

- How was it for you?
- Do you have any questions?
