# Supplementary material for: Delirium in German Nursing Homes – a qualitative study of care practice from the perspective of nurses and general practitioners
Source: BMC Geriatr. 2026 May 5;26:634. doi: 10.1186/s12877-026-07592-7 (PMC13141596; doi:10.1186/s12877-026-07592-7)
Supplement: Supplementary file 3 — Supplementary Material 3. [file 12877_2026_7592_MOESM3_ESM.docx]

# DeliA: Delirium in Nursing Homes

Study-ID:

**Sociodemographic and professional biographical data checklist: Nurses**

1. Gender:
2. Year of birth:
3. Workplace:
   - Nursing Home
   - Temporary employment agency
   - Others:
4. Current position (multiple responses possible):
   - Nursing service management
   - Residental area management
   - Certified Nurse
   - Trainee:
   - Others:
5. Vocational training/ University degree (multiple responses possible):
   - Registered Nurse
   - Nursing Assistant
   - Geriatric Nurse
   - Geriatric Nursing Assistant
   - Nursing B.A./B.Sc.
   - Nursing M.A./M.Sc.
   - Others:
6. Completed specialist training cours(es):
7. How long have you been working in your profession since graduating? years
8. How long have you been in your current position? (see 3.) ____ years
9. What is your current employment percentage? ___ %
10. How many people work in care sector at your facility?
11. How many people work in your area / on your ward in nursing?
12. Does your area/ward have a specific focus? If so, what is it? (e.g. dementia)
13. How many residents do you care for on average per shift?
14. How often do you notice these residents experiencing short-term confusion with personality changes (delirium)?
    - never
    - rarely
    - frequently
    - always
