# Supplementary material for: Delirium in German Nursing Homes – a qualitative study of care practice from the perspective of nurses and general practitioners
Source: BMC Geriatr. 2026 May 5;26:634. doi: 10.1186/s12877-026-07592-7 (PMC13141596; doi:10.1186/s12877-026-07592-7)
Supplement: Supplementary file 4 — Supplementary Material 4. [file 12877_2026_7592_MOESM4_ESM.docx]

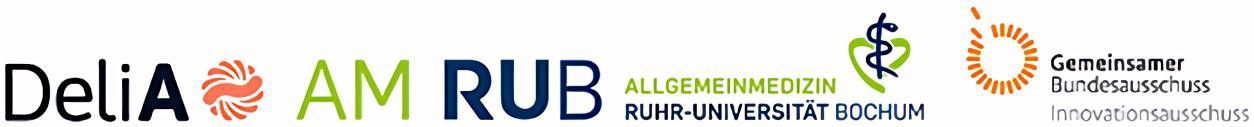


# DeliA: Delirium in Nursing Homes

Study-ID:

**Sociodemographic and professional biographical data checklist: General Practitioners**

| □ Female |
| --- |
| - Male - Gender divers |

1. Gender:
2. Year of birth:
3. Workplace:
   - Practice
     - Solo practice
     - Group practice
     - Shared pracitce facilities
     - Medical care center
   - Hospital
   - Nursing home
4. Specialist medical training:
5. How long have you been working in your profession? _____ years
6. What is your current employment percentage? ___ %
7. How many patients do you treat on average per quarter?
8. How often do you care for patients in nursing homes per quarter?
   - Number of facilities:
   - Number of visits per facility:
   - Number of patients per facility:
9. How many patients experience delirium?
